# Effects of daily full‐term infant skin‐to‐skin contact on behavior and cognition at age three – secondary outcomes of a randomized controlled trial

**DOI:** 10.1111/jcpp.13679

**Published:** 2022-08-09

**Authors:** Nicole Rheinheimer, Roseriet Beijers, Nina Bruinhof, Kelly H. M Cooijmans, Carolina de Weerth

**Affiliations:** ^1^ Department of Cognitive Neuroscience, Donders Institute for Brain, Cognition and Behaviour Radboud University Medical Center Nijmegen The Netherlands; ^2^ Department of Social Development, Behavioural Science Institute Radboud University Nijmegen The Netherlands

**Keywords:** Skin‐to‐skin contact, term‐birth, behavioral development, executive functioning

## Abstract

**Background:**

Daily skin‐to‐skin contact (SSC) during early infancy fosters the long‐term development of children born preterm. This is the first randomized controlled trial assessing the potential beneficial effects of daily SSC on executive functioning and socio‐emotional behavior of children born full‐term. Whether children of mothers who experienced prenatal stress and anxiety benefitted more from SSC was also explored.

**Methods:**

Pregnant women (*N* = 116) were randomly assigned to a SSC or care‐as‐usual (CAU) condition. Women in the SSC condition were instructed to perform one hour of SSC daily from birth until postnatal week five. Prenatal stress was measured with questionnaires on general and pregnancy‐specific stress and anxiety completed by the mothers in gestational week 37. At child age three, mothers filled in questionnaires on children's executive functioning, and externalizing and internalizing behavior. Analyses were performed in an intention‐to‐treat (ITT), per‐protocol, and dose–response approach. Netherlands Trial Register: NL5591.

**Results:**

In the ITT approach, fewer internalizing (95% CI = 0.11–1.00, *U* = 2148.50, *r* = .24, *p* = .001) and externalizing (95% CI = 0.04–2.62, *t* = 2.04, *d* = 0.38, *p* = .04) problems were reported in the SSC condition compared to the CAU condition. Multivariate analyses of variance did not show group differences on executive functioning. Additional analyses of covariance showed no moderations by maternal prenatal stress.

**Conclusions:**

Current findings indicate that early daily SSC in full‐term infants may foster children's behavioral development. Future replications, including behavioral observations of child behavior to complement maternal reports, are warranted.

## Introduction

For preterm infants, skin‐to‐skin contact (SSC) during hospitalization results in positive outcomes, such as increased cardiorespiratory and thermal regulation, fewer infections, faster weight gain, enhanced sleep, and decreased crying behavior (Feldman, Rosenthal, & Eidelman, 2014; Kostandy & Ludington‐Hoe, 2019). Several studies have shown that benefits of SSC extend to full‐term infants. For instance, in full‐term infants, the practice of SSC immediately after delivery is related to improved cardiovascular stability, weight gain, sleep, as well as decreased crying behavior (Ionio, Ciuffo, & Landoni, 2021; Moore, Bergman, Anderson, & Medley, 2016). While assessments of SSC in full‐term infants have largely been restricted to the hours after delivery, research on preterm infants indicates that SSC is beneficial beyond the first postnatal hours. When performed daily throughout preterm infants' first postnatal weeks or month, SSC has been related to improved long‐term cognitive and behavioral development (Feldman et al., 2014). The current paper reports results of the first randomized controlled trial (RCT) to investigate effects of daily SSC in full‐term children on cognitive and behavioral outcomes in early childhood.

During SSC, the naked infant is placed on the mother's bare chest (World Health Organization, 2003). The precise mechanisms underlying the effects of SSC on infants are mainly unknown (Ionio et al., 2021). However, it is suggested that the exchange of sensory cues during SSC (i.e., touch, warmth, odor, vocalizations) has regulating effects on the infant's physiology. For instance, SSC immediately decreases infants' levels of the stress hormone cortisol, and increases the release of the hormone oxytocin (Beijers, Cillessen, & Zijlmans, 2016; Vittner et al., 2018). Additionally, repeated SSC facilitates face‐to‐face interactions, and allows mother and infant to familiarize with each other's interactive cues, hereby fostering the development of reciprocal interaction patterns (Moore et al., 2016). These positive reciprocal mother–infant interactions can, in turn, benefit infant regulation of the neuro‐endocrine system (Nagasawa, Okabe, Mogi, & Kikusui, 2012; Norholt, 2020; Vittner et al., 2018). In general, it is thought that repeated SSC might facilitate the development of neuro‐biobehavioral systems early in life, which, in turn, foster development throughout childhood (Moberg, Handlin, & Petersson, 2020).

As mentioned before, longitudinal studies show beneficial effects of SSC in the first postnatal month on child outcomes later in life (Moore et al., 2016). Studies on preterm infants linked the practice of daily SSC to improved cognitive functioning, including executive functioning, across childhood and beyond (Charpak et al., 2017; Feldman et al., 2014; Ropars, Tessier, Charpak, & Uriza, 2018). Additionally, studies on preterm infants also showed that SSC can benefit children's behavioral development. Charpak et al. (2017) reported that preterm infants receiving daily SSC displayed fewer externalizing problems (e.g., hyperactivity, aggressiveness, socio‐deviant conduct) at age 20. No effects were found on internalizing problems (e.g., social problems, withdrawal, and anxiety). However, another study on preterm infants reported that SSC facilitated children's reciprocity during conversations with their mother at age ten (Feldman et al., 2014). Likewise, the only longitudinal study to date on daily SSC with full‐term infants reported enhanced engagement and reciprocity during a mother–child conversation on emotional memories at age nine (Bigelow & Power, 2020).

However, these previous findings on full‐term infants were restricted to the assessment of a mother–child conversation, and additionally, this study was not an RCT (Bigelow & Power, 2020). Moreover, mothers in this study were requested to perform up to 6 hrs of SSC a day. This long period of SSC requires a large time investment, and may hamper implementation of SSC into daily routines for some mothers. The current RCT is the first to study long‐term effects of SSC on the development of children born full‐term. We report secondary outcomes of an intervention consisting of a five‐week period in which mothers of full‐term infants were asked to perform one daily hour of SSC. Specifically, we assessed whether SSC benefits three‐year‐olds' executive functioning, as well as externalizing and internalizing behavior. Previous assessments of this RCT found beneficial effects of SSC on breastfeeding duration (Cooijmans, Beijers, Brett, & de Weerth, 2021).

Studies often report relations between maternal stress and anxiety during pregnancy and compromised offspring behavioral and cognitive development (Graignic‐Philippe, Dayan, Chokron, Jacquet & Tordjman, 2014; van den Bergh et al., 2020). However, prenatal psychosocial stress may not only increase offspring's vulnerability for poorer outcomes later in life, but also offspring's plasticity, making them more susceptible to early postnatal circumstances, for better and for worse (Beijers et al., 2020). This enhanced plasticity would increase offspring's vulnerability to negative experiences, but also increase their susceptibility to positive experiences in the postnatal period (Graignic‐Philippe et al., 2014). Therefore, we additionally explored whether children of mothers with increased prenatal psychosocial stress benefitted more from the SSC intervention in terms of cognitive and behavioral development than children of mothers with lower prenatal psychosocial stress.

## Methods

### Trial design

This RCT consisted of two groups (SSC intervention vs. care‐as‐usual). The primary aim was to test the effectiveness of SSC in decreasing maternal postpartum depressive symptoms (not reported here). This study examines secondary outcomes of a follow‐up assessment at age three. The baseline assessment of this RCT was registered at the Netherlands Trial Register (Trial‐ID: NL5591), according to CONSORT guidelines. The trial protocol was also published (Cooijmans, Beijers, Rovers, & de Weerth, 2017). All assessments of this RCT were approved by the ethics committee of the Faculty of Social Sciences at Radboud University (Baseline: ECSW2015‐2311‐358; Follow‐up: SW2017‐1303‐497).

### Participants

Pregnant women (*N* = 116) were recruited in Nijmegen, the Netherlands, through flyers, social media, and a participant database. Inclusion criteria were: singleton pregnancy, no use of drugs, fluent in Dutch, ≥18 years old, no severe physical/mental health issues, and no ongoing participation in other studies. Infants' inclusion criteria at birth were: born full‐term (≥37 weeks), birthweight ≥2,500 g, no congenital anomalies, and an Apgar score of ≥7 at 5 min post‐birth.

### Randomization and masking

During recruitment, a cover story was used. Pregnant women were informed that the study investigated associations between infant sleep and feeding, the role of mother–infant contact, as well as physical and mental health of mother and infant. They were also told that a subgroup would perform a daily contact‐period throughout the first 5 weeks after delivery. An independent researcher performed computer‐generated randomization to the care‐as‐usual (CAU) or SSC condition (1:1), with random blocks of four and six, stratified by parity (multiparae or primiparae). Randomization was stored individually in sealed envelopes.

### Procedure

Interested women were visited at home between gestational week 34 and 36. They received further information in accordance with the cover story, gave written informed consent, filled in questionnaires on demographics, as well as prenatal stress and anxiety, and were assigned to a group. Women in the SSC condition were additionally instructed to practice SSC for 1 hr a day for 5 weeks, starting immediately after birth. From birth, mothers of both conditions filled in daily physical contact‐logbooks, including information on the amount of SSC, holding, and breastfeeding performed. Debriefing took place at a follow‐up visit after 1 year. Another follow‐up assessment took place around the children's third birthday, including online questionnaires on their children's cognition and behavior.

### Measures

For all outcome variables, internal consistency was assessed using Revelle's omega total (ω_*t*_, Revelle & Condon, 2018). Internal consistency estimates >.70 are considered adequate for questionnaire‐based group comparisons (Nunnally & Bernstein, 1994).

#### Maternal prenatal stress and anxiety

During the prenatal home‐visit, women filled in four questionnaires on pregnancy‐specific, as well as general stress and anxiety. The State Anxiety Scale of the State–Trait Anxiety Inventory (ω_*t*_ = .91; STAI; Van der Ploeg, Defares, & Spielberger, 1981) measures general state anxiety with 20 questions on a four‐point scale, for which a sum score is computed. Pregnancy‐specific anxiety was measured with a sum score of the Pregnancy‐Related Anxiety Questionnaire (ω_*t*_ = .90; PRAQ; van den Bergh, 1990), which contains 34 questions on anxiety experienced during pregnancy on five‐point scales. Daily hassles were measured with the Alledaagse Problemen Lijst (ω_*t*_ = .79; APL; Vingerhoets, Jeninga, & Menges, 1989), containing 49 questions addressing general stressful events. Participants indicated whether an event had occurred in the past 2 months, and how affected they had been by it on four‐point scales. Scores of how much the hassles affected mothers were summed up. The Pregnancy Experience Scale (ω_*t*_ = .90; PES; DiPietro, Ghera, Costigan, & Hawkins, 2004) measured pregnancy‐specific stress. On 43 items, participants indicated whether a situation was an uplift and/or hassle, on two four‐point scales. Ratio scores were computed per participant, dividing the sum score of uplifts (ω_*t*_ = .92) by that of hassles (ω_*t*_ = .85).

A single grand composite ‘Maternal prenatal stress’ (ω_*t*_ = .90) was created by standardizing and averaging the four questionnaires (Beijers et al., 2020). If one questionnaire was missing, an average was computed across the other three. If more than one questionnaire was missing, no composite was computed for that participant and their score on maternal stress was considered missing.

#### Skin‐to‐skin contact (SSC)

The mother–infant physical contact‐logbook was used to track periods of holding, breastfeeding, or SSC, in five‐minute intervals during the first five postnatal weeks. Maternal holding and breastfeeding were not counted as SSC. Moreover, SSC and holding by other people were reported in the logbook, but were not counted toward mother–infant SSC. Mothers in both conditions filled in the logbook every two to three hours throughout the day, on a moment that suited them well during their daily routine (e.g., after feeding or diaper changes). The amount of SSC performed a day was only computed if at least 80% of that day was filled, and if logbooks were filled in sufficiently (≥21 of 35 days). In total, 90 mothers (CAU = 41; SSC = 49) had filled in the logbook sufficiently. For valid logbooks, missing days were replaced with the dyad's mean amount of SSC of two days before and after. The total amount of SSC performed throughout the intervention period was only computed for logbooks with sufficient data.

#### Children's executive functioning at age three

The Behavior Rating Inventory of Executive Function‐Preschool (BRIEF‐P) examined everyday executive functioning with 63 items on three‐point scales (Sherman & Brooks, 2010). The questionnaire contained five subscales: Flexibility (ω_*t*_ = .92), Inhibition (ω = .91), Emotion Regulation (ω_*t*_ = .86), Planning and Organizing (ω_*t*_ = .73), and Working Memory (ω_*t*_ = .87). Higher scores on the BRIEF‐P indicated more difficulties. While an overall score of executive functioning is commonly computed for the BRIEF‐P in older children, Skogan et al. (2016) have demonstrated that this unidimensional conceptualization is not adequate at age three. In young children, different components of executive functioning develop at differing paces (Anderson, 2002). We therefore included the five BRIEF‐P subscales in the analyses.

#### Children's problem behavior at age three

Mothers reported on their children's internalizing and externalizing behavior in two questionnaires. The first questionnaire, the Dutch version of the Child Behavior Checklist/1.5–5, contained 99 items on five‐point Likert scales (CBCL; Achenbach & Rescorla, 2000). The CBCL factor Internalizing (ω_*t*_ = .76) included the subscales emotionally reactive, anxious/depressed, somatic complaints, and withdrawal. The CBCL factor Externalizing (ω_*t*_ = .92) contained the subscales attention problems, and aggressive behavior. Higher scores on the CBCL indicated more problem behavior. Mothers also filled in the Strengths and Difficulties questionnaire (SDQ; Goodman, 1997). The SDQ contained 25 items (10 reversed) on three‐point scales. The SDQ factor Internalizing (ω_*t*_ = .65) consisted of the subscales emotional symptoms, and peer problems. The factor Externalizing (ω_*t*_ = .71) consisted of prosocial behavior, and hyperactivity. Due to relatively low internal consistency, the SDQ was not included in further analyses.

### Missing data

**Figure 1:**
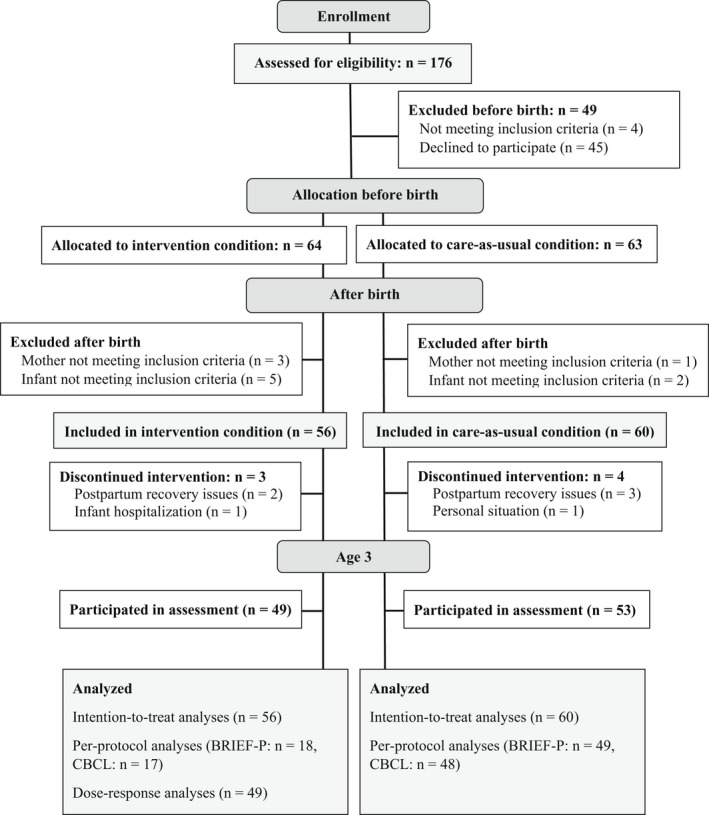
Participant flowchart

Of all 116 mothers (CAU = 60; SSC = 56), four mothers in the CAU and three mothers in the SSC condition discontinued the intervention (see Figure 1). Of the 104 mothers (CAU = 53; SSC = 49) participating in the three‐year follow‐up, the BRIEF‐P was incomplete for two mothers in the SSC and four mothers in the CAU condition. Five mothers in the SSC and five in the CAU condition did not complete the CBCL. Prenatal questionnaires STAI and PRAQ were missing for one mother in the SSC condition. The APL was missing for one mother in the CAU condition. The PES was missing for two mothers in the SSC and two mothers in the CAU condition. Composite scores on prenatal stress were missing for one mother in the SSC and one in the CAU condition.

### Statistical analyses

#### Statistical approaches

All analyses were conducted in R version 4.1.1 (R Core Team, 2020). Similar to previous assessments of this RCT (Cooijmans et al., 2021), current analyses were performed with three approaches. In the intention‐to‐treat (ITT) approach, all dyads were included in the analyses, regardless of compliance (CAU = 60; SSC = 56). Missing values on moderators and outcome variables were imputed with the expectation–maximization method (Liu & Brown, 2013). In the per‐protocol (PP) approach, dyads of both conditions were only included if they had no missing outcome data on the BRIEF‐P (CAU = 49; SSC = 18) and the CBCL (CAU = 48; SSC = 17). Dyads of the SSC condition were included in the PP approach if they had complete logbooks (>60% filled in) and if they had performed at least 1 hr of SSC on at least 28 of the 35 days (i.e., 80% of the days). This 80% criterion is based on a prior study that asked mothers to perform SSC for 4 weeks (Bigelow, Power, MacLellan‐Peters, Alex, & McDonald, 2012). The exploratory dose–response (DR) approach was performed within the SSC condition, including only mothers with valid logbooks (SSC = 49). In DR analyses, the total duration of SSC was used as a continuous predictor, and missing outcome values were imputed.

#### Preliminary analyses

Sample size calculations for the primary study outcome (maternal depressive symptoms) indicated that, accounting for attrition, 116 dyads suffice to detect a medium effect size (*f* = 0.24) with a power of 80% (Cooijmans et al., 2017). Outliers of the BRIEF‐P subscales and CBCL factors were winsorized (replaced with the mean plus/minus three times the standard deviation; Tukey, 1977). Differences in baseline characteristics and study variables were assessed for the ITT and PP approaches, using independent sample t‐tests for normally, and Mann–Whitney *U* tests for non‐normally distributed continuous data. For categorical data, χ^2^ tests were used. For the DR approach, Pearson correlations were computed (‘*stats*’, R Core Team, 2020).

### Main analyses

#### Children's executive functioning

Group differences on executive functioning were assessed with five subscales of the BRIEF‐P, in a one‐way multivariate analysis of variance (MANOVA). In case of group differences on baseline characteristics, the variable was corrected for, using a multivariate analysis of covariance (MANCOVA). The interaction of maternal prenatal stress with condition was assessed in an additional MANCOVA (‘*car*’, Fox & Weisberg, 2019).

#### Children's problem behavior

In case of group differences in baseline characteristics, two analyses of covariance (ANCOVA) were performed, one on internalizing and one on externalizing behavior. If no group differences were indicated, we referred to the outcomes of previously described *t*‐tests, Mann‐Whiney *U* tests, and Pearson correlations to answer our research question. The interaction of maternal prenatal stress with condition was assessed in two additional ANCOVAs.

## Results

Participants were recruited from April 2016 until September 2017. The follow‐up assessment took place between September 2019 and August 2020. The participant flow is presented in Figure 1. No study‐related harms were reported.

### Preliminary analyses

Outliers were identified on the following variables: BRIEF‐P subscales Flexibility (*N* = 1), Inhibition (*N* = 1) and Regulation (*N* = 1); CBCL Internalizing (*N* = 2); prenatal questionnaires: PES (*N* = 1), STAI (*N* = 1), APL (*N* = 1), and the composite of prenatal stress (*N* = 1). Group comparisons of baseline characteristics and study variables are listed in Table 1. The intervention condition performed significantly more SSC than the CAU condition. On average, mothers in the SSC condition performed 58 min (*SD* = 26 min) and the CAU condition 12 min (*SD* = 23 min) of SSC a day throughout the intervention phase. Across the intervention period, mothers in the SSC condition provided approximately between 42 and 83 min of daily SSC whereas mothers in the CAU condition provided between zero and 60 min (for a day‐by‐day graph see Cooijmans et al., 2021). There was no significant difference between primiparae and multiparae women in the amount of SSC performed (*t* = 0.37, *d* = 0.08, *p* = .72). Of all mothers in the SSC condition, 18 performed sufficient daily SSC for PP analyses (>60 min on at least 28 of the 35 days). Correlations between outcome variables are reported in Table 2. Pearson correlations of the total amount of SSC with the outcome variables for the DR approach were insignificant.

**Table 1:** Descriptive statistics and group comparisons for mother–infants dyads of the three‐year follow‐up assessment in the skin‐to‐skin contact (SSC) and care‐as‐usual (CAU) conditions

	Intention‐to‐treat^a^		Per‐protocol	
	CAU (*N* = 60)	SSC (*N* = 56)	SSC (*N* = 18)	
	*M* (*SD*)	*M* (*SD*)	*M* (*SD*)	Statistic^g^
Baseline characteristics^a^				
Maternal age (years)	32.48 (3.05)	32.36 (3.85)	32.90 (3.80)	478.00^b^
Maternal educational level	6.87 (1.79)	6.82 (1.55)	6.78 (1.48)	564.00^b^
Smoking (% No)	100.00	96.43	97.87	.33^c^
Alcohol (% No)	100.00	98.21	97.87	.33^c^
C‐section (% No)	94.80	92.70	97.87	.00^c^
Birth order (%)				
First	46.70	48.21	33.33	1.25^c^
Second	38.33	19.64	38.89	
Third	15.00	19.64	27.78	
APGAR score	9.70 (0.62)	9.84 (0.42)	9.72 (0.58)	474.50^b^
Child sex (% girls)	43.33	58.93	61.11	1.25^c^
Birthweight (grams)	3567.47 (358.77)	3650.05 (414.93)	3760.56 (454.59)	−1.59^d^
GA at birth (weeks)	40.02 (1.10)	40.08 (1.01)	40.16 (1.03)	−0.51^d^
Age at follow‐up (years)	3.02 (0.12)	3.03 (0.12)	3.02 (0.10)	437.50^b^

	*M* (*SD*)	*M* (*SD*)	Statistic	*M* (*SD*)	Statistic
Total SSC (min.)^a^	308.17 (442.41)	2067.68 (850.65)	147.50***^b^	2905.90 (497.52)	18.00***^b^
Maternal prenatal stress^e^ ^,^ ^f^	−0.09 (0.85)	0.10 (1.14)	1569.00^b^	−0.06 (0.92)	305.00^b^
PRAQ^a^	−0.01 (0.96)	−0.02 (1.05)	1257.50^b^	−0.05 (0.89)	453.00^b^
STAI State^a^	−0.16 (0.83)	0.15 (1.14)	1037.00^b^	−0.01 (1.00)	456.50^b^
PES^a^	−0.08 (0.88)	0.07 (1.11)	1160.00^b^	−0.10 (0.92)	427.50^b^
APL^a^	−0.08 (0.93)	0.09 (1.08)	1144.50^b^	−0.06 (0.62)	474.00^b^

Outcome variables^e^	*M* (*SD*)	*M* (*SD*)	Statistic	*M* (*SD*)	Statistic
BRIEF‐P					
Flexibility	3.91 (2.63)	3.42 (2.77)	1887.50^b^	6.22 (5.44)	514.50^b^
Inhibition	8.40 (5.59)	7.12 (4.26)	1872.50^b^	7.78 (5.54)	483.00^b^
Memory	6.94 (4.62)	5.65 (4.30)	1952.50^b^	5.83 (4.81)	477.00^b^
Planning	4.77 (2.79)	4.02 (2.49)	1927.00^b^	4.06 (2.75)	488.50^b^
Regulation	5.61 (3.85)	4.05 (3.00)	2104.50*^b^	3.94 (2.69)	563.00^b^
CBCL Internalizing	10.65 (1.18)	10.10 (1.05)	2148.50**^b^	10.09 (0.90)	497.00^b^
CBCL Externalizing	17.83 (3.88)	16.51 (3.10)	2.04*^d^	16.24 (3.19)	1.79^d^

GA, gestational age; *M*, mean; *SD*, standard deviation.

^a^ *M* and *SD* are presented for non‐imputed data.

^b^ Mann‐Whitney *U* tests for non‐normally distributed data.

^c^ χ^2^ tests for categorical data.

^d^ Independent samples t‐tests for normally distributed data.

^e^ *M* and *SD* for winsorized and imputed data.

^f^ Standardized data for all moderators.

^g^ Comparing the per‐protocol sample with the CAU sample.

*p < .05; **p < .01; ***p < .001.

**Table 2:** Pearson correlations among study variables across the entire sample

Variable	BRIEF‐P^a^					CBCL^a^		Moderator^a^
	Flexibility	Inhibition	Memory	Planning	Regulation	CBCL Int.	CBCL Ext.	Pre. stress
Flexibility	–	–	–	–	–	–	–	–
Inhibition	.20*	–	–	–	–	–	–	–
Memory	.20*	.72***	–	–	–	–	–	–
Planning	.14	.64***	.72***	–	–	–	–	–
Regulation	.40***	.43***	.35***	.36**	–	–	–	–
CBCL Int.	.57***	.44***	.40***	.40**	.56***	–	–	–
CBCL Ext.	.23*	.78***	.60***	.44***	.58***	.59***	–	–
Pre. Stress	.22*	.20*	.25**	.23*	.16	.13	.08	–
Total SSC^b^	.05	−.08	−.01	.08	−.04	.05	−.23	.17

Pre. Stress, maternal prenatal stress.

^a^ Winsorized and imputed data. Higher scores indicate more difficulties.

^b^ Correlations with total amount of SSC for the dose–response approach within the intervention condition (*N* = 49).

*p < .05; **p < .01; ***p < .001.

### Main analyses

#### Children's executive functioning

Assumptions of multivariate normality for the MANOVAs on the five BRIEF‐P subscales were not met. The dependent variables were therefore square root transformed, and Pillai's Trace is reported as a robust statistic (Ateş, Kaymaz, Kale, & Tekindal, 2019). There were no significant differences between conditions on executive functioning (Table 3).

**Table 3:** Multivariate Analyses of Variance (MANOVA) on executive functioning and exploratory analyses of covariance of maternal prenatal stress on executive functioning and behavior

	Intention‐to‐treat				Per‐protocol				Dose–response^a^			
**Main analysis**												
*BRIEF‐P* ^b^	*V*	η^2^	*F*(*5,110*)	*p*	*V*	η^2^	*F*(*5,65*)	*p*	*V*	η^2^	*F*(*5,45*)	*p*
Condition	.043	.043	0.95	.450	.057	.057	0.74	.594	.023	.023	0.19	.965
**Exploratory analyses**												
*BRIEF‐P* ^b^	*V*	η^2^	*F*(*5,108*)	*p*	*V*	η^2^	*F*(*5,62*)	*p*	*V*	η^2^	*F*(*5,45*)	*p*
Condition	.048	.067	1.17	.329	.069	.068	0.85	.518	.024	.040	1.19	.965
Pren.	.125	.126	3.00	.014	.190	.190	2.73	.028	.181	.181	1.72	.153
Cond. × Pren.	.019	.022	0.47	.799	.036	.036	0.43	.825	.157	.157	1.45	.229
*Internalizing* ^b^		η^2^	*F*(*1,112*)	*p*		η^2^	*F*(*1,60*)	*p*		η^2^	*F*(*1,45*)	*p*
Condition		.071	7.43	.007		.071	2.92	.093		.000	0.125	.725
Pren.		.042	4.93	.029		.076	0.28	.278		.132	6.835	.012
Cond. × Pren.		.016	1.76	.187		.002	0.09	.761		.014	0.630	.432
*Externalizing*		η^2^	*F*(*1,112*)	*p*		η^2^	*F*(*1,60*)	*p*		η^2^	*F*(*1,45*)	*p*
Condition		.042	4.17	.043		.045	2.75	.102		.077	2.68	.109
Pren.		.030	3.50	.064		.008	0.47	.495		.078	3.82	.057
Cond. × Pren.		.003	0.33	.567		.007	0.44	.512		.010	0.47	.495

*V*, Pillai's trace for MANOVAs; η^2^, partial eta^2^; Cond., Condition; Pren., prenatal stress.

^a^ Dose–response analyses within intervention condition with duration of skin‐to‐skin as continuous predictor.

^b^ Dependent variables were square‐root transformed.

MANCOVAs testing the interaction of condition with prenatal stress on the BRIEF‐P subscales were insignificant (Table 3).

#### Children's problem behavior

**Figure 2:**
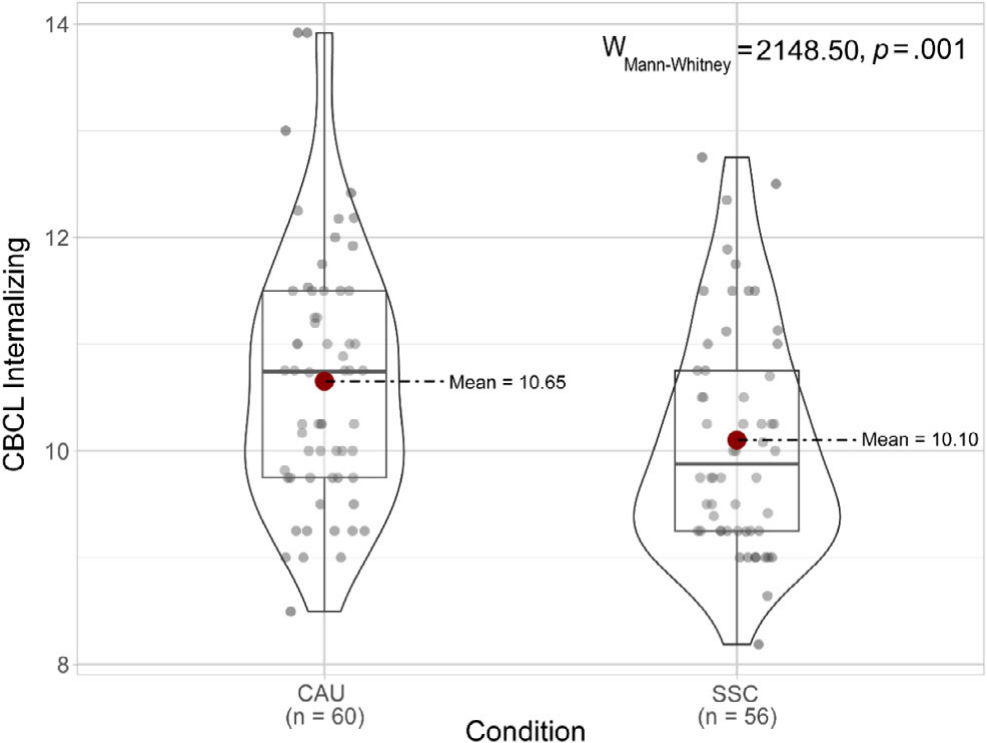
Means, standard deviations, and distributions of scores on Internalizing behavior for the CAU and SSC condition for the intention‐to‐treat approach [Color figure can be viewed at wileyonlinelibrary.com]

**Figure 3:**
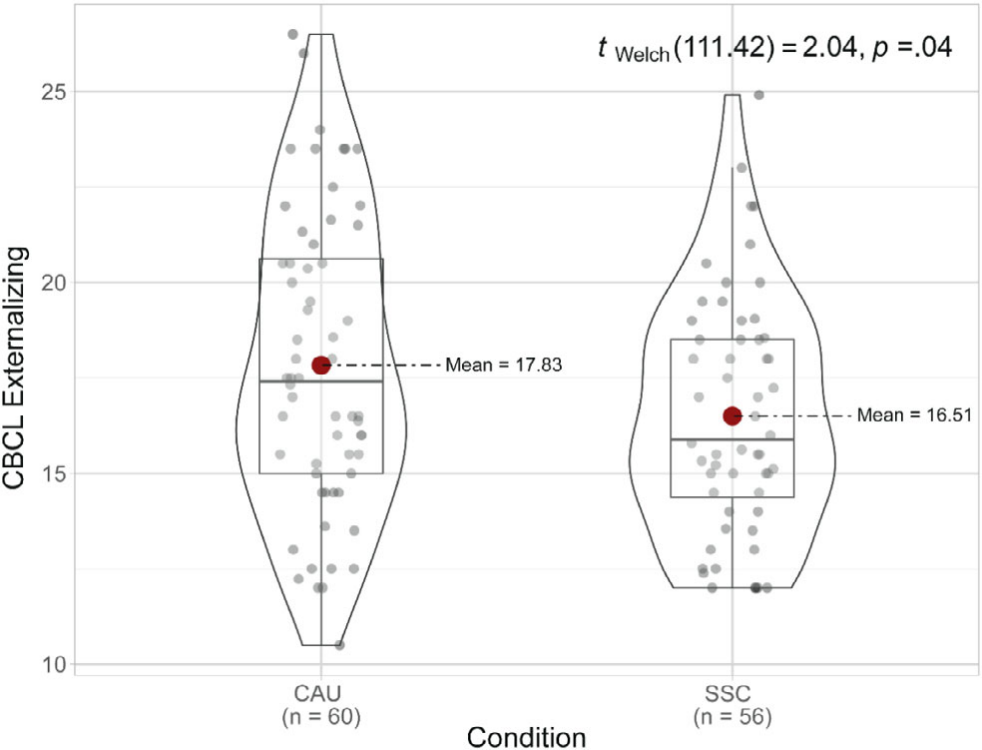
Means, standard deviations, and distributions of scores on Externalizing behavior for the CAU and SSC condition for the intention‐to‐treat approach [Color figure can be viewed at wileyonlinelibrary.com]

Since there were no significant group differences in baseline characteristics, and as such no need to control for variables, we could rely on the group differences as reported in Tables 1 and 2. Group differences in internalizing and externalizing problems for the ITT approach are visualized in Figures 2 and 3.

In the ITT approach, a Mann–Whitney *U* test on internalizing problems was significant (95% CI = 0.11–1.00, *U* = 2148.50, *r* = .24, *p* = .01). The SSC condition (Mdn = 9.88, *M* = 10.10, *SD* = 1.05) showed fewer internalizing problems compared to the CAU condition (Mdn = 10.74, *M* = 10.65, *SD* = 1.18). An independent samples *t*‐test showed significantly fewer externalizing problems (95% CI = 0.04–2.62, *t* = 2.04, *d* = 0.38, *p* = .04) in the SSC condition (*M* = 16.51, *SD* = 3.10), compared to the CAU condition (*M* = 17.83, *SD* = 3.88) in the ITT approach. No significant group differences were found in the PP (Table 1) and DR approaches (Table 2).

ANCOVAs testing the interaction of condition with prenatal stress on internalizing and externalizing behavior were insignificant (Table 3).

## Discussion

This RCT investigated whether skin‐to‐skin contact (SSC) with full‐term infants during the first five postnatal weeks affected executive functioning and child behavior problems 3 years later. Additionally, we explored whether the intervention was more beneficial for children of mothers who had experienced prenatal stress and anxiety, compared to those of mothers who had not. In the intention‐to‐treat analyses, children of the SSC condition showed fewer internalizing and externalizing behavior problems than children of the CAU condition. No group differences were found on executive functioning. Maternal prenatal symptoms did not moderate the effects of SSC on executive functioning and behavior problems. Lastly, no significant results were found in per‐protocol and dose–response analyses.

Beneficial effects of SSC on children's behavior, as reported in the intention‐to‐treat analyses, are in line with findings in preterm infants (Charpak et al., 2017; Feldman et al., 2014). The only previous study to date on long‐term outcomes of SSC for full‐term infants reported beneficial effects of SSC on children's behavior during a mother–child conversation (Bigelow & Power., 2020). However, this study was not an RCT and mothers were not blind to the study goal during recruitment, potentially introducing a sampling bias to the study. The current RCT recruited mothers with a cover story, and significant effects on behavior were found in the fully randomized sample. Therefore, these findings constitute substantial evidence that in full‐term infants, just as in preterm infants, early SSC may benefit their behavioral development.

We also performed per‐protocol analyses (PP), including mothers of the SSC condition only if they had performed the requested hour of SSC regularly. However, only 18 mothers had performed sufficient SSC, and we did not find significant effects despite the PP means being virtually identical to the ITT means (see Table 1), potentially due to a lack of power. We also did not find dose–response effects of the amount of SSC performed within the SSC condition, which possibly indicates that shorter durations of SSC might suffice to achieve desired effects on child behavior. DR effects of SSC have, however, been found on breastfeeding duration, indicating that increased amounts of SSC might benefit other important outcomes for infant and mother (Cooijmans et al., 2021).

The current absence of an effect on EF is not in line with literature on preterm infants. For example, a previous study on preterm children reported facilitating effects of daily SSC on EF throughout childhood (Feldman et al., 2014). However, to our knowledge, no studies have assessed the effects of SSC on EF in full‐term infants, and SSC possibly may not have large effects on EF in infants born full‐term. Preterm infants' cognitive development may benefit more from SSC because they are generally more fragile, their neurodevelopment is strained, and they are deprived of physical contact because of their need of incubator care (Norholt, 2020).

Another reason for the current null‐findings on EF might be the chosen assessment age. We assessed children at age three, while the previous study on preterm infants reported effects on EF at age 5 and 10 years (Feldman et al., 2014). It is suggested that EF undergoes crucial developmental shifts after age three, and therefore EF assessments are more reliable later in childhood (Anderson, 2002; Garon, Piccinin, & Smith, 2016). Potentially, effects on EF in our study may not yet be visible. Additionally, the current assessment relied on parental report, while previous effects of SSC on preterm infants' EF were assessed through a cognitive task (Feldman et al., 2014). Parental report and experimental tasks on EF have been suggested to be incongruent (Garon et al., 2016), and future research should therefore combine parental reports with cognitive tasks. Combining these measures may additionally rule out the possibility of maternal response biases.

Finally, our low intervention compliance may have played a role, as higher SSC intervention compliance has been reported in preterm infants (Charpak et al., 2017; Feldman et al., 2014), where the intervention is usually integrated into hospital care (Blomqvist, Frölund, Rubertsson, & Nyqvist, 2013). Implementation of the intervention into daily home routines may be challenging for mothers of full‐term infants. Also, mothers in the current study were blind to the intervention aims. In preterm infants, mothers are aware of the potential of SSC, and might therefore be more engaged.

The current study has substantial strengths. This is the first RCT assessing long‐term benefits of SSC in full‐term infants, and drop‐out rate was considerably low throughout the study. However, limitations should be noted. First, the current cohort was largely homogeneous, including mainly families of high SES and education. Second, mothers were debriefed when their child turned one. This might have influenced maternal reports on child EF and behavior at age three. Although this cannot entirely be ruled out, we consider it unlikely, since biased maternal assessments would have caused similar effects on EF reporting. Lastly, the restricted sample size in the current study did not allow for an assessment of potential variables that may mediate the effects of daily SSC on child outcomes, hence revealing the underlying working mechanisms. This is an important next step to pursue in future research in larger study populations.

## Conclusion

This study indicates that daily SSC in full‐term infants' first postnatal month may help prevent behavioral problems 3 years later. Additionally, previous assessments of this RCT demonstrated beneficial effects on breastfeeding duration (Cooijmans et al., 2021). Taken together, the current RCT contributes substantially to the evidence of SSC effects on children born full‐term. This RCT hopefully motivates further research on daily SSC interventions with healthy full‐term children. Future studies should address ways of enhancing parental intervention compliance, and combine questionnaire‐based assessments with behavioral observations.
